# A New Triterpene From *Uncaria macrophylla* and Its Antitumor Activity

**DOI:** 10.3390/molecules17021883

**Published:** 2012-02-14

**Authors:** Guangli Sun, Xiaopo Zhang, Xudong Xu, Junshan Yang, Mingliang Zhong, Jingquan Yuan

**Affiliations:** 1 Institute of Medicinal Plant Development, Chinese Academy of Medical Sciences & Peking Union Medical College, Beijing 100193, China; Email: guanglisun@126.com (G.S.); xiaopozhang2011@126.com (X.Z.); junshanyang@sina.com (J.Y.); mingliangzhongsky@163.com (M.Z.) ; yjqgx@163.com (J.Y.); 2 Department of Pharmacy, School of Pharmacy, Hebei United University, Tangshan, Hebei 063000, China; 3 Guangxi Institute of Medicinal Plant Development, Nanning, Guangxi 530023, China

**Keywords:** *Uncaria macrophylla*, Rubiaceae, 3β, 6β, 19α-trihydroxy-12-oleanen-28-oic acid, antitumor activity

## Abstract

On our ongoing investigation, a new oleanolic triterpene, 3β,6β,19α-trihydroxy-12-oleanen-28-oic acid (**1**) was obtained from the chloroform-soluble portion of the 90% alcohol-water extract of the stem bark of *Uncaria macrophylla*. Its structure was elucidated by extensive spectroscopic methods, including 1D and 2D (^1^H-^1^H COSY, HSQC and HMBC) NMR and HR-ESI-MS. The cytotoxicities of the compound was evaluated against two cancer cell lines of MCF-7 and HepG2 by the MTT method, and the compound exhibited weak activities with the IC_50_ values of 78.2 µg/mL and 73.9 µg/mL.

## 1. Introduction

**Figure 1:**
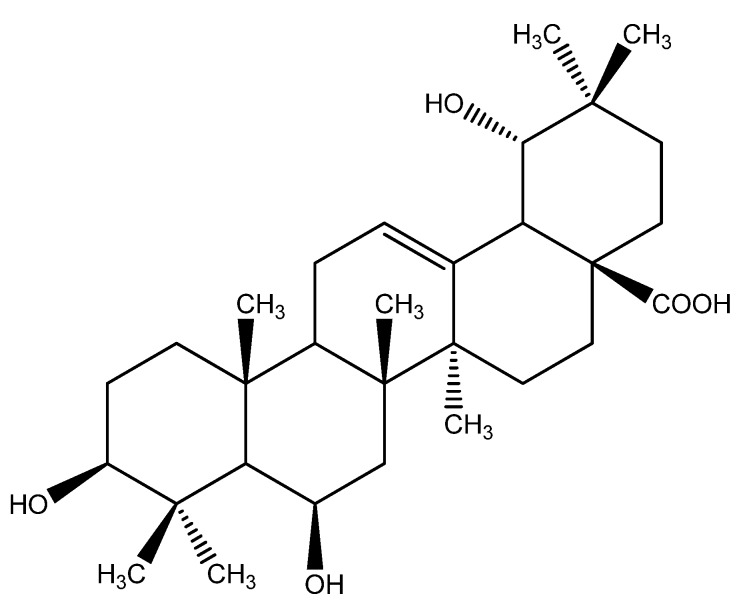
Structure of compound **1**.

The genus *Uncaria* is widely distributed in tropical regions, including Southern Asia, Africa and South America [1], and most plants of the genus *Uncaria* have been used as important sources of medicinal natural products in the family of Rubiaceae. Many species of the genus *Uncaria* have been used for curing fevers, nervous disorders, apasmolytic, analgesic, hypertension [2], cancer, arthritis, diabetes, inflammation [3,4,5]. Previous studies show that alkaloids, triterpenes and flavones [6,7,8] are the most widespread of the secondary metabolites isolated from the genus *Uncaria*. As a part of our systematic isolation of phytochemical constituents, a new triterpene (**1**, Figure 1) was isolated from the chloroform-soluble fraction of the alcohol-water extract of *U . macrophylla*. In this paper, the isolation, structure elucidation by extensive spectral methods including 1D, 2D NMR and MS and the antitumor activities are presented for the first time.

## 2. Results and Discussion

### 2.1. Structural Identification

Compound **1** (Figure 1) was isolated as a white solid from the chloroform extracts of *Uncaria macrophylla*. The HR-ESI-MS spectrum revealed a molecular ion peak at 511.3383 [M+Na]^+^ (calculated 511.3399) corresponding to the molecular formula of C_3__0_H_48_O_5_Na.

The ^1^H-NMR peaks at δ 1.29 (3H, s), 1.27 (3H, s), 1.16 (3H, s), 1.07 (3H, s), 1.05 (3H, s), 0.97 (3H, s) and δ 0.94 (3H, s) were due to seven methyl groups at quaternary carbons. In the ^13^C-APT spectrum, signals of the methyl groups at δ 17.31, 25.21, 17.76, 18.66, 28.51, 25.45 and 28.84 were clearly observed. An oleﬁnic proton signal at δ 5.35 in ^1^H-NMR spectrum along with signals at 125.36 and 144.10 in the ^13^C-NMR spectrum suggested compound **1** was an oleanane pentacyclic triterpene posessing a Δ^12, l3^ moiety [9]. Furthermore, signals at 182.65 in the ^13^C-NMR spectrum together with the IR absorption at 1699.17 cm^−1^ indicated the presence of an carboxyl group in **1**. The ^1^H-NMR signals at δ 4.92 and δ 3.26 together with the ^13^C-NMR spectral data at δ 80.39, 69.22 and 82.75 suggested compound **1** to be a trihydroxy substituted pentacyclic triterpene [10]. 

**Figure 2:**
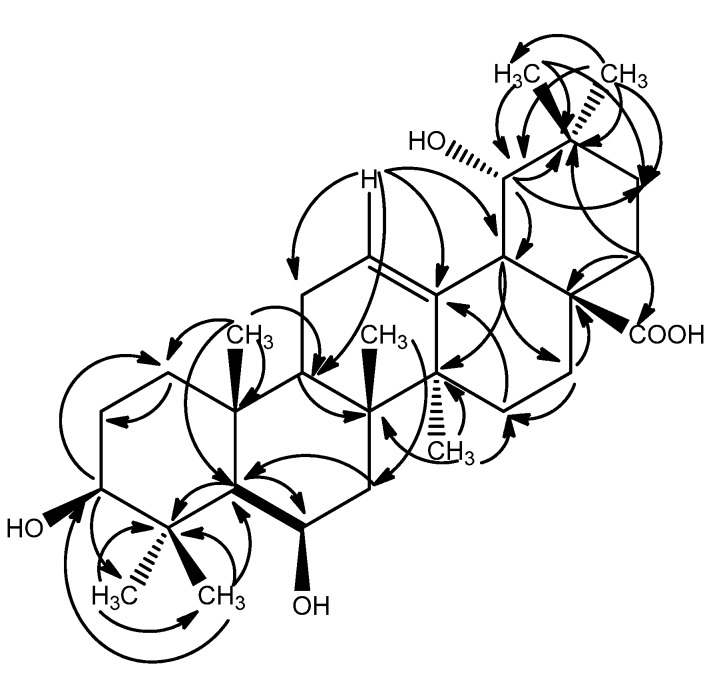
Selected HMBC (^2^*J* and ^3^*J*) correlations (H→C) and H-H COSY correlations of compound **1**.

The Δ^12, l3^ structure was confirmed by the HMBC correlations between H-12 (δ 5.35) and C-9 (δ 49.83), C-11 (δ 24.90), C-13 (δ 144.10), C-18 (δ 45.42). The HMBC cross-peaks between H-3 (δ 3.09) and C-1 (δ 41.96), between H_3_-25 and C-1 (δ 41.96), C-5 (δ 57.68), C-9 (δ 49.83), C-10 (δ 38.04), and the evidence from the chemical shift and the *J* value of the axial proton at C-3 (δ 3.09, dd, *J* = 11.4, 3.6Hz, H-3) suggested the hydroxy at C-3 accepted an β configuration. The H-HCOSY correlation between H-5 (δ 0.75) and H-6 (δ 4.49), along with the HMBC correlation between H-5 (δ 0.75) and C-6 (δ 69.22) indicated a hydroxyl group was attached to C-6 (Figure 2) [10]. 

**Figure 3:**
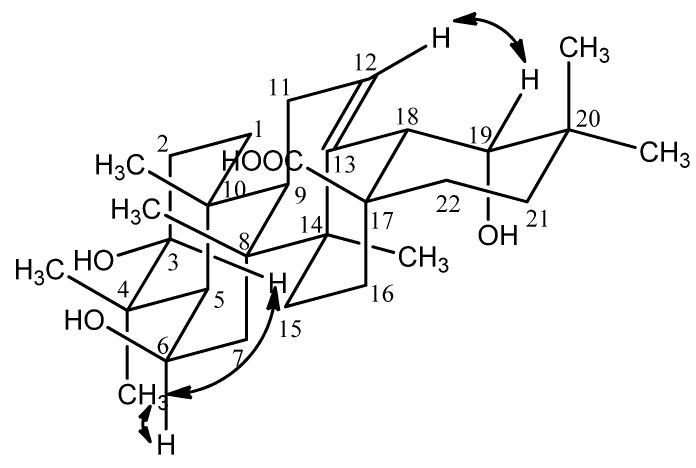
Selected NOESY correlations of compound **1**.

The relative configuration of hydroxyl group at C-6 was deduced to be β by the observed cross-peaks of H-3 (δ 3.09) and H_3_-23 (δ 1.05), H-6 (δ 4.49) and H_3_-23 (δ1.05) in the NOESY experiment (Figure 3). 

The hydroxyl group at the 19 position induced a downfield shift of the resonance of the axial proton at C-16. This signal resonated at δ 2.26 and was observed as a peak of td with *J* = 13.2, 3.6 Hz supporting the stereochemistry of 19-OH to be α and being compatible only with a *cis* stereochemistry of the ring D/E junction, the proton at δ 2.26 was conﬁrmed to be H-16 [11]. The appearance of characteristic signals at δ 2.52 (lH, s, H-18) in the ^1^H-NMR spectrum and at δ 73.82 ppm (C-19, quaternary carbon) in the ^13^C-NMR spectrum suggested that **1** was an oleanane acid derivative with a hydroxyl group attached to C-19, which was confirmed by the observed HMBC correlations between H-18 (δ 3.07) and C-14 (δ 43.24), C-16 (δ 28.88), between H-19 (δ 3.26) and 45.42 (C-18), 36.24 (C-20), 29.63 (C-21), 25.45 (C-29), between H_3_-29 (δ 0.97) and 82.75 (C-19), 36.24 (C-20), 29.63 (C-21), 28.84 (C-30), between H_3_-30 (δ 0.94) and 82.75 (C-19), 36.24 (C-20), 29.63 (C-21), 25.45 (C-29) (Figure 2). Furthermore, the NOESY correlations between H-12 (δ 5.35) and H-19 (δ 3.31) confirmed that the 19-OH accepted an α-configuration (Figure 3).

Consequently, the structure **1** was established and determined to be 3β,6β,19α-trihydroxy-12-oleanen-28-oic acid (Figure 1), and it was named uncarioleanic acid. Compound **1** was evaluated for its inhibitory ability against two cancer cell lines (MCF-7 and HepG2) *in vitro*. The results showed that compound **1** was weakly effective in inhibiting the growth of MCF-7 and HepG2, with IC_50_ values against the two cell line of 78.2 µg/mL and 73.9 µg/mL while those of cisplatin were 7.5 µg/mL and 8.2 µg/mL, respectively.

## 3. Experimental

### 3.1. General

The NMR spectra were recorded by Bruker AV-600 spectrometer (600 MHz for ^1^H and 150 MHz for ^13^C), using CD_3_OD as solvent and tetramethylsilane (TMS) as internal standard. Infrared spectra were measured using FTIR-8400S spectrometer. The HR-ESI-MS data were obtained on the Micross Mass Autospec-Ultima ETOF mass spectrophotometer (Agilent, Milford, MA, USA). Ultraviolet spectra were recorded in MeOH on a Shimadzu UV-160A, UV-Visible Recording Spectrophotometer. Chromatography was performed on silica gel (200–300 mesh, Qingdao Haiyang Chemical Factory, Qingdao, China) and ODS gel (40–60 μm, Daiso Co., Ltd., Japan).

### 3.2. Plant Material

The medicinal material was collected from Guangxi Province in 2010, and identified by Dr. Jing Quan Yuan at Key Laboratory of Bioactive Substances and Resources Utilization of Chinese Herbal Medicine, Ministry of Education, Institute of Medicinal Plant Development, Chinese Academy of Medical Sciences & Peking Union Medical College. A voucher specimen with No. 20101132 has been deposited in.

### 3.3. Extraction and Isolation

The air-dried and powdered sample (5 kg) was extracted successively with 90% EtOH-H_2_O (50 kg, 70 °C, 2 hours). The extracts were dried under reduced pressure using a rotary evaporator to yield petroleum ether (32.3 g), chloroform (31.8 g), ethyl acetate (15.6 g) and water (8.5 g) extracts. The chloroform extract was chromatographed over a silica gel column using a stepwise gradient system petroleum ether-acetone. The eluted fraction A (petroleum ether/acetone 70:30, 3.2 g) was then subjected to a Sephadex LH 20 column chromatography using chloroform-methanol (40:60), the obtained fraction D (160 mg) was subjected to reverse chromatography (ODS) with a gradient of MeOH-H_2_O (35:65) to get compound **1** (8 mg).

### 3.4. Spectral Data

*3β,6β,19α-Trihydroxy-12- oleanen-28-oic acid* (**1**). White solid. [α]^25^_ D_ – 2.67°, IR ν_max_ (cm^−1^): 3455, 2933, 1699. ESI-MS m/z: [M+Na]^ +^ 511.3383. For ^1^H-NMR and ^13^C-NMR (CD_3_OD) spectra, see Table 1. 

**Table 1:** ^1^H-NMR (600 MHz, CD_3_OD) and ^13^C-NMR (150 MHz, CD_3_OD) data for (**1**).

Position	^1^H(δ)	^13^C(δ)	HMBC
1	1.54 (2H, m)	41.96	28.19 (C-2), 80.39 (C-3), 57.68 (C-5), 38.04 (C-10), 17.31 (C-25)
2	1.51(1H, m),	28.19	41.96 (C-1)
	1.68 (1H, m)		
3	3.09 (1H, dd, *J* =11.4, 3.6 Hz)	80.39	41.96 (C-1), 17.76 (C-24)
4		40.93	
5	0.75 (1H, d, *J* = 1.8 Hz)	57.68	80.39 (C-3), 40.93(C-4),
			69.22 (C-6), 38.04 (C-10)
6	4.49 (1H, br.s)	69.22	
7	0.97(1H, m),	42.01	57.68 (C-5), 40.12 (C-8),
	1.54 (1H, m)		43.24 (C-14)
8		40.12	
9	3.31 (1H, s)	49.83	42.01 (C-7), 40.12 (C-8),
			17.31 (C-25)
10		38.04	
11	2.02 (2H, m)	24.90	49.83 (C-9), 38.04 (C-10),
			144.10 (C-13)
12	5.35 (1H, br. s)	125.36	24.90 (C-11), 144.10 (C-13),
			45.42 (C-18)
13		144.10	
14		43.24	
15	0.99 (1H, m),	29.83	144.10 (C-13)
	1.70 (1H, m)		
16	1.57 (1H, m), 2.26 (1H, td, *J* = 13.2, 3.6 Hz)	28.88	29.83 (C-15), 46.98 (C-17)
17		46.98	
18	3.07 (1H, d, *J* = 3.6 Hz)	45.42	43.24 (C-14), 28.88 (C-16)
19	3.26 (1H, d, *J* = 3.6 Hz)	82.75	45.42 (C-18), 36.24 (C-20),
			29.63 (C-21), 25.45 (C-29)
20		36.24	
21	0.99 (1H, m),	29.63	
	1.70 (1H, m)		
22	1.60 (1H, m),	34.27	46.98 (C-17), 36.24 (C-20),
	1.76 (1H, m)		182.65 (C-28)
23	1.05 (3H, s)	28.51	80.39 (C-3), 40.93 (C-4),
			57.68 (C-5)
24	1.16 (3H, s)	17.76	80.39 (C-3), 40.93 (C-4),
			57.68 (C-5), 28.51 (C-23)
25	1.29 (3H, s)	17.31	57.68 (C-5), 40.12 (C-8),
			49.83 (C-9), 38.04 (C-10)
26	1.07 (3H, s)	18.66	42.01 (C-7), 40.12 (C-8),
			49.83 (C-9), 43.24 (C-14)
27	1.27 (3H, s)	25.21	29.83 (C-15)
28		182.65	
29	0.97 (3H, s)	25.45	82.75 (C-19), 36.24 (C-20),
			29.63 (C-21), 28.84 (C-30)
30	0.94 (3H, s)	28.84	82.75 (C-19), 36.24 (C-20),
			29.63 (C-21), 25.45 (C-29)

### 3.5. Bioassays

Antitumor activity was assayed on MCF-7 and HepG2 cells using cisplatin as positive control. Cells were plated in the appropriate media on 96-well plates in a 100 μL total volume at a density of 6 × 104 cells/mL. The final concentrations of each compound were 0.625, 1.25, 2.5, 5.0, 10 μg/mL. The plates were incubated at 37 °C in 5% CO_2_ for 72 h. Cell viability was determined based on the mitochondrial conversion of MTT to formazan.

## 4. Conclusions

A new triterpene, 3β,6β,19α-trihydroxy-12-oleanen-28-oic acid (**1**) was isolated from the stem bark of *U. macrophylla*. The isolation of the new compound was a new addition to the molecular diversity of *U. macrophylla*. Compound **1** exhibited weak antitumor activity. 
